# SARS‐CoV‐2 infection in lung transplant recipients induces circulating exosomes with SARS‐CoV‐2 spike protein S2

**DOI:** 10.1002/ctm2.576

**Published:** 2021-11-04

**Authors:** Sandhya Bansal, Sofya Tokman, Timothy Fleming, Gabriel N. Maine, Kristina Sanborn, Ramsey Hachem, Ankit Bharat, Michael A. Smith, Ross M. Bremner, T. Mohanakumar

**Affiliations:** ^1^ Norton Thoracic Institute St. Joseph's Hospital and Medical Center Phoenix Arizona; ^2^ Department of Pathology and Laboratory Medicine Beaumont Health Royal Oak Michigan; ^3^ Department of Medicine Washington University School of Medicine St. Louis Missouri; ^4^ Department of Surgery Northwestern University Chicago Illinois

Dear Editor,

This is a single center study to demonstrate the importance of exosomes in developing a more sensitive method to detect severe acute respiratory syndrome coronavirus 2 (SARS‐CoV‐2) infection from symptomatic lung transplant recipients (LTxRs) along with symptomatic and asymptomatic patients waiting for lung transplant (LTx). These exosomes carrying SARS‐CoV‐2 spike protein can be immunogenic to mice.

World Health Organization declared SARS‐CoV‐2 a global pandemic in March 2020.^1^ This virus caused coronavirus disease 2019 (COVID‐19), which has killed over 4.8 people worldwide to date. Diagnosis of COVID‐19 is commonly established by detecting the SARS‐CoV‐2 within respiratory secretions using polymerase chain reaction (PCR) assays.^2^, ^3^ For the patients with end‐stage respiratory disease, LTx is the last treatment option.^3^ In our previous reports, we have shown exosomes containing viral antigens in LTxRs with respiratory viral infections and this may play a role in the pathogenesis of chronic lung allograft dysfunction.^4^ We demonstrate that exosomes carry SARS‐CoV‐2 spike protein S2 in symptomatic and asymptomatic LTxR individuals waiting for LTx, suggesting that detection of exosomes with SARS‐CoV‐2 spike protein S2 can be developed as more sensitive approach for detecting SARS‐CoV‐2 infection.

We analyzed exosomes from plasma of 27 LTxRs‐COVID‐19 and 57 asymptomatic patients (Table S1) (Asx) for the presence of exosomes carrying the SARS‐CoV‐2 spike and nucleocapsid protein (December 2019 to March 2021). The results demonstrated the presence of SARS‐CoV‐2 spike antigen S2 and nucleocapsid on exosomes from all symptomatic LTxRs (Figure 1A, Table S2A). In addition, 6/6 patients positive for SARS‐CoV‐2 infection by RT‐PCR (Figure 2B, Table S2) and 16/57 (28.0%) asymptomatic patients waiting for LTx (Asx) also had exosomes positive for the SARS‐CoV‐2 spike protein S2 and nucleocapsid protein (Figure 2C, Table S2A). The specificity toward SARS‐CoV‐2 was confirmed by Western blot analysis using specific antibodies (Abs) (Figure 3A, B). Exosomes containing SARS‐CoV‐2 spike protein S2 from 7 patients from symptomatic and asymptomatic groups were also tested for other proteins. These exosomes demonstrated the presence of other peptides including angiotensin II receptor type 1 (AGTR1), which has suggestive role in viral entry into the cells in addition to proinflammatory responses,^5^ macrophage stimulating 1 (MST1), MST1/2 is a mediator of the innate immune response, eliciting macrophage phagocytosis and cytokines,^6^ Granzyme‐B (GRA‐B), which plays an important role in immune regulation and cytotoxicity of cells in response to viral infections.^7^ In addition, there is also presence of lung self‐antigens (SAgs) (Kα1 Tubulin [Kα1T], Collagen V [Col‐V]) and nuclear factor kappa B (NFkB) (Figure 2A, B and Table S2B). AGTR1, GRA‐B and MST1 were elevated in plasma exosomes from LTxRs‐COVID‐19 and in Asx (*p* < .05). Presence of these peptides indicates an active or recent SARS‐CoV‐2 infection.

**FIGURE 1:**
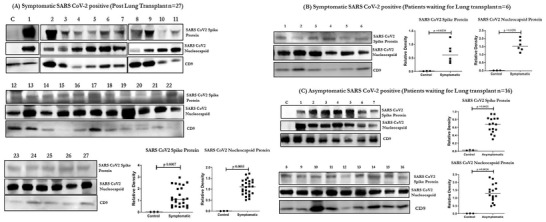
Western blot and densitometry of exosomal proteins from LTxRs with known SARS‐CoV‐2 infection (LTxRs‐COVID‐19; *n* = 27) for SARS‐CoV‐2 spike protein S2 and Nucleocapsid protein, C is the control and 1–27 are exosome samples from each patient. (B) Western blot and densitometry of exosomal proteins from control and from Symptomatic LTx candidates (*n* = 6) for SARS‐CoV‐2 spike protein S2 and Nucleocapsid protein. (C) Western blot and densitometry of exosomal proteins from control and from asymptomatic LTx candidates (Asx; *n* = 16) for SARS‐CoV‐2 spike protein S2 and Nucleocapsid protein. *Only Upper band in the blot corresponds to SARS‐CoV‐2 spike protein in (A) and (B). *All statistical analyses were performed using Mann–Whitney Test using GraphPad Prism version 8.0.

**FIGURE 2:**
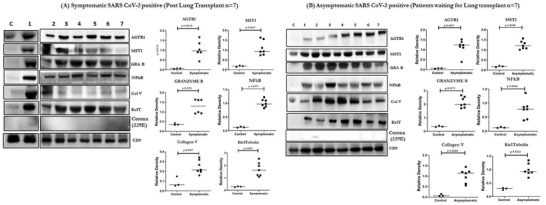
Western blot of exosome proteins from LTxRs with known SARS‐CoV‐2 (LTxRs‐COVID‐19; *n* = 7) for MST1, AGTR1, GRA‐B, and coronavirus proteins (229E). C is the control and 1–7 are exosome samples from each patient. (B) Densitometry and statistical analysis of exosomes from control and LTxRs‐COVID‐19, which was positive for SARS‐CoV‐2 spike protein S2, MST1, AGTR1, GRA‐B, lung SAgs (Col‐V, Kα1T), and NFkB proteins. (C) Western blot of exosome proteins from control and from asymptomatic LTx candidates (Asx; *n* = 7) for MST1, AGTR1, GRA‐B, coronavirus protein (229E), lung SAgs (Col‐V, Kα1T), and NFkB. (D) Densitometry and statistical analysis from control and Asx plasma positive for the SARS‐CoV‐2 spike protein S2, MST1, AGTR1, GRA‐B, coronavirus protein (229E), lung SAgs (Col‐V, Kα1T), and NFkB proteins. *All statistical analyses were performed using Mann–Whitney test using GraphPad Prism version 8.0

**FIGURE 3:**
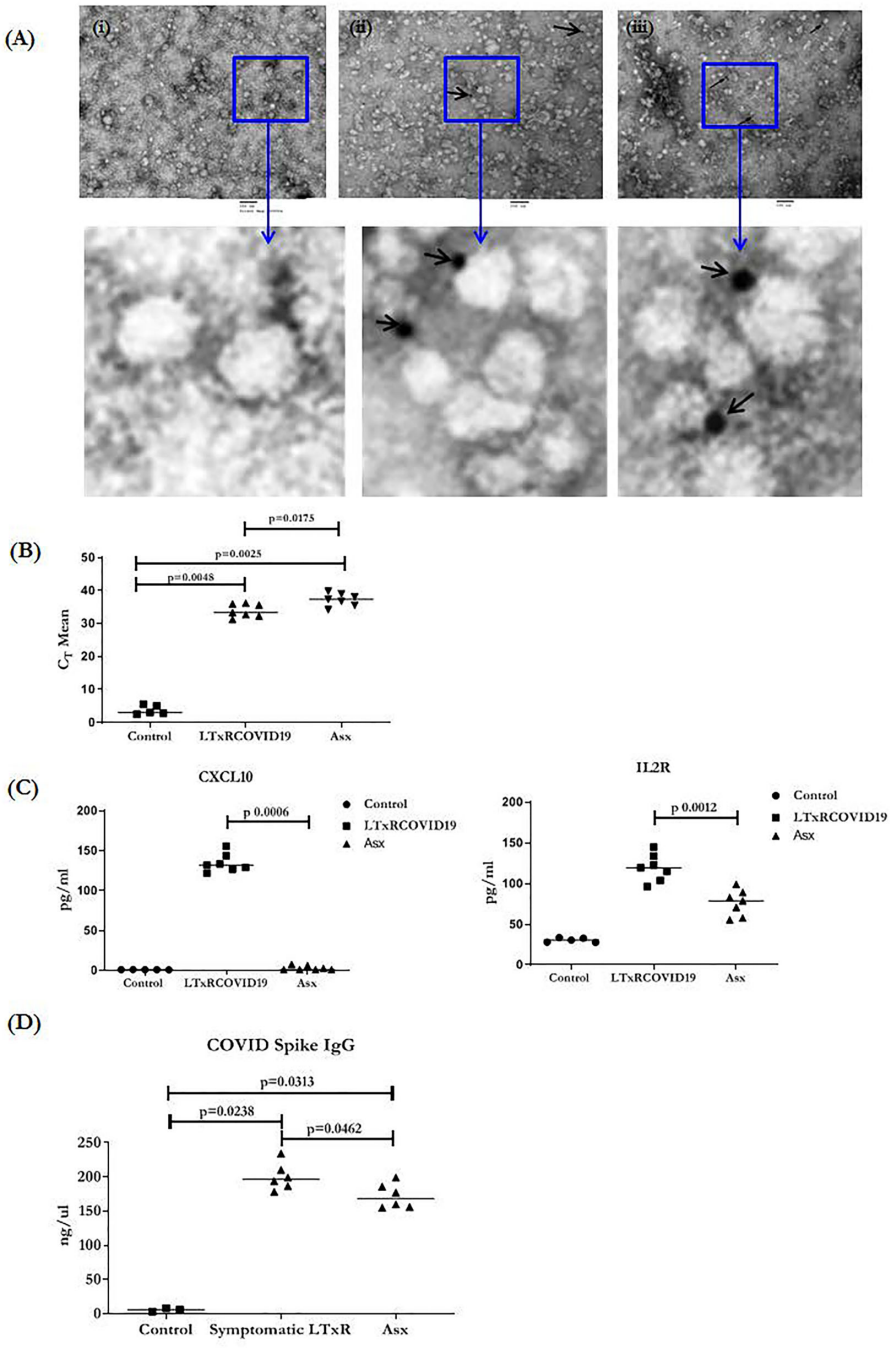
TEM images of exosomes from LTxRs with known SARS‐CoV‐2 infection (LTxRs‐COVID‐19) and from asymptomatic LTx candidates (Asx). One each (i) negative control (ii) exosomes positive for SARS‐CoV‐2 spike protein from sample 1 (iii) exosomes positive for nucleocapsid protein from sample 2. Magnified views of images are given in panel below. Scale = 100 nm. (B) RT‐PCR of SARS‐CoV‐2 from exosomes of LTxRs‐COVID‐19 (*n* = 7) and Asx (*n* = 7). Data are presented as cT Mean. (C) Cytokine profiling data of LTxRs‐COVID‐19 (*n* = 7) and Asx (*n* = 7). Data are presented as pg/ml. (D) ELISA of SARS‐CoV‐2 spike antibody from mice at D28. Mice were immunized with COVID spike antigen positive exosomes isolated from LTxRs (LTxRs‐COVID‐19) and SARS‐CoV‐2 spike antigen positive exosomes isolated from SARS‐CoV‐2 RT‐PCR negative Asx. *All statistical analyses were performed using Mann–Whitney test using GraphPad Prism version 8.0

Exosomes containing SARS‐CoV‐2 spike protein S2 was further confirmed by mass spectroscopy and transmission electron microscopy (TEM). Desired protein bands were extracted from SDS–PAGE of positive exosomes and subjected to mass spectroscopy. Bands from two samples were aligned with peptides of SARS‐CoV‐2 spike protein and nucleoprotein: (a) LPDDFTGCVIAWNSNNLDSKVGGNYNYLYRLFRK with UNIPORT ID PODTC2 and molecular weight 141.1 kDa and (b) WYFYYLGTGPEAGLPYGANK with UNIPORT ID PODTC9 and molecular weight 45.6 kDa. TEM and immunostaining of the exosomes using Abs specific to SARS‐CoV‐2 spike protein S2 and nucleocapsid protein revealed the presence of the spike protein S2 and nucleocapsid antigen on the surface of exosomes (Figure 3A). Mass Spectroscopy and TEM results are in agreement to the Western blot results. Cytokine analysis of plasma from LTxRs‐COVID‐19 and Asx, demonstrated increased levels of proinflammatory cytokines (IL12, RANTES, IP10, IFNγ, Eotaxin, IL2R, and MCP1) but increase in IP10 and IL2R cytokines were statistically significant Figure 3B. Elevated IP10 levels may correlate with severity of disease as increased levels of IP10 were identified in LTxRs‐COVID‐19 but not in the Asx (*p* = .0006). IL2R was also significantly higher in the plasma from LTxRs‐COVID‐19 than in plasma from Asx (*p* = .0012).^8^ SARS‐CoV‐2 RNA was also present in exosomes from Asx and LTxRs‐COVID‐19 at significantly higher levels as compared to controls samples Figure 3C.

Sera collected on day 28 following immunization C57BL/6 mice with exosomes^9^ carrying SARS‐CoV‐2 spike antigen from LTxRs‐COVID‐19 and Asx patients resulted in development of Abs to SARS‐CoV‐2 spike protein as shown in Figure 3D.

## CONCLUSION

In conclusion, our study demonstrates that exosomes isolated from plasma of LTxRs‐COVID‐19 and a proportion of end stage lung disease patients waiting for LTx have SARS‐CoV‐2 spike protein antigen S2. 16/57 asymptomatic patients waiting for LTx also had exosomes with SARS‐CoV‐2 spike protein S2. Exosomes with SARS‐CoV‐2 spike protein S2 also contained AGTR1, MST1, GRA‐B, and NFkB.

**FIGURE 4:**
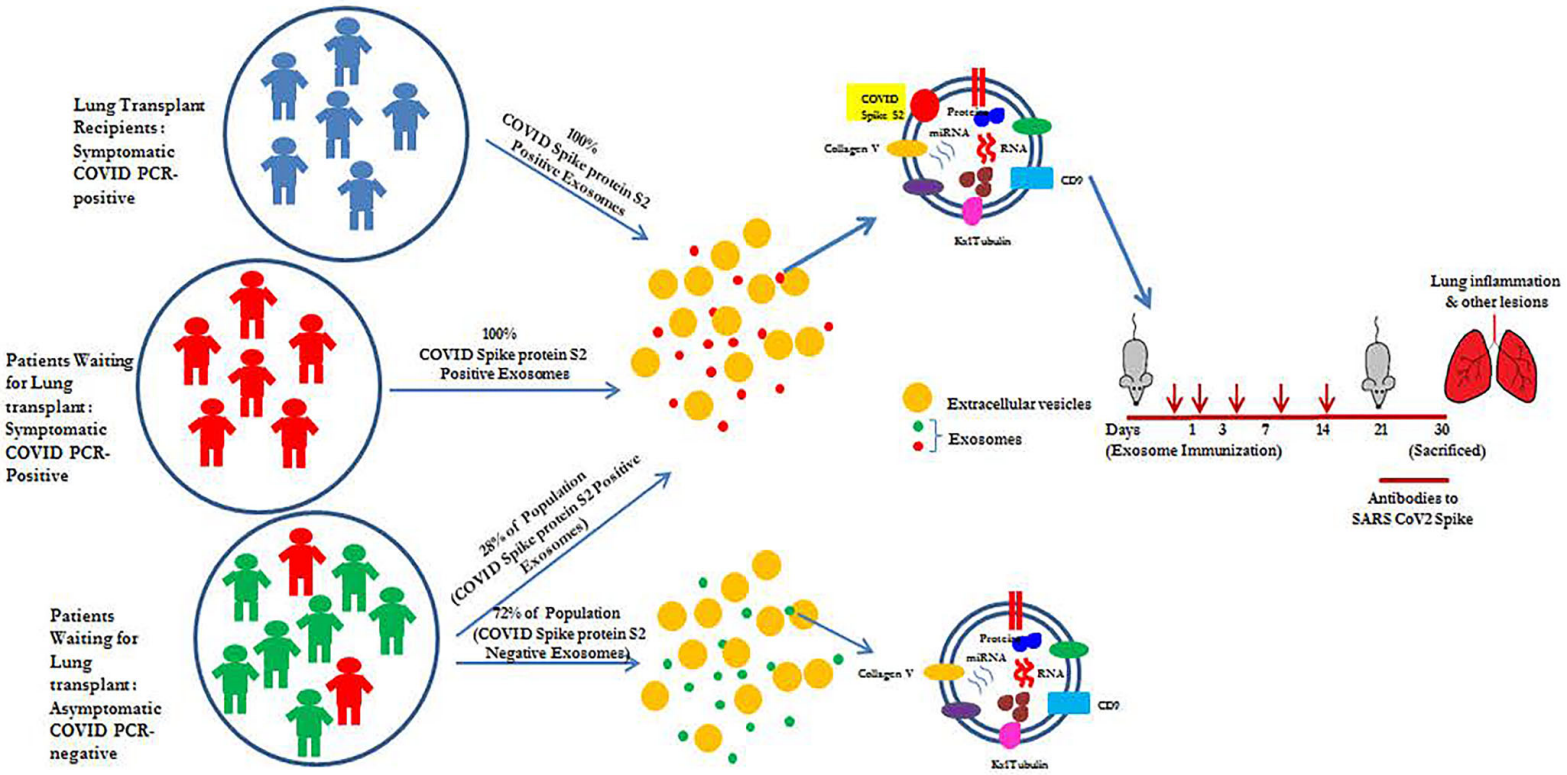
Model demonstrating the findings symptomatic LTxRs and symptomatic patients waiting for LTx and proportion of asymptomatic patients (waiting for LTx) carry spike protein S2 on the exosomes. Immunizing the mice with exosomes from both groups carrying exosomes with spike protein induced Abs to SARS‐CoV‐2 spike protein mice

Mass spectroscopy and TEM, with specific Abs, demonstrated that exosomes contain SARS‐CoV‐2 spike protein S2 and nucleocapsid protein. Immunization of mice with exosomes carrying spike protein S2 induced Abs specific to SARS‐CoV‐2 spike protein S2 indicating not only the specificity but also immunogenicity of exosomes. SARS‐CoV‐2 spike protein S2 in the exosomes also suggests that the exosomes acquire the spike protein antigen during active infection. Exosomes may provide a more sensitive assessment of active viral infection than the SARS‐CoV‐2 by PCR due to pieces of nonactive viral genes which are amplified by the current PCR approach (false positivity). Additional studies are needed to confirm this finding. Summary of our findings are given in Figure 4.

Limitation of our study includes lack of serial samples. Our studies have shown 16/57 PCR negative patients waiting LTx also had exosomes with SARS‐CoV‐2 spike protein S2 demonstrating that exosomes may provide an additional tool for diagnosis of SARS‐CoV‐2 infection.

## CONFLICT OF INTEREST

The authors declare they have no competing interests. All authors have reviewed and approved the manuscript and have contributed in a substantial and intellectual manner to the work.

## Supporting information

Supporting Information

Supporting Information
