# Supplementary material for: SARS‐CoV‐2 infection in lung transplant recipients induces circulating exosomes with SARS‐CoV‐2 spike protein S2
Source: Clin Transl Med. 2021 Nov 4;11(11):e576. doi: 10.1002/ctm2.576 (PMC8567032; doi:10.1002/ctm2.576)
Supplement: Supplementary file 1 — Supporting Information [file CTM2-11-e576-s002.docx]

**MATERIALS AND METHODS**

**1.1 Patient cohort and demographics**

We performed a retrospective study of 5 SARS-CoV-2 negative controls, 27 adult LTxRs diagnosed as SARS-CoV-2 positive, 6 adult patients waiting for LTx were PCR positive with SARS-CoV-2 infection and 57 adults waiting for LTx since December 2019-March 2021 were asymptomatic and PCR negative. All the patients were eligible for the study and they had undergone LTx at St. Joseph’s Hospital, Phoenix, Arizona and plasma samples were available for the analysis. This study was approved by the Institutional Review Board at St. Joseph’s Hospital (IRB# PHXB16-0027-10-18). Patient demographics, transplant details, and laboratory data collected from patient charts are given in **Table S1.**

**1.2 Exosome isolation and nanoparticle tracking analysis**

Exosomes were isolated from 500µl of plasma obtained from all the group of patients using Invitrogen Exosome Isolation kit followed by 0.22-micron filtration. All exosomes were checked for size by NanoSight NS300 (Malvern, Great Malvern, UK) and the mean size of the particles used in our experiments was <200 nm.^8^ Nanosight images are provided in **Figure S1**.

**1.3 Characterization of exosomes using western blot**

Total exosome protein (15µg) was resolved by polyacrylamide gel electrophoresis and the proteins were transferred onto a polyvinylidene difluoride membrane. Exosomes from all the patients (pool of exosomes from 5 SARS-CoV-2 negative controls, SARS-CoV-2 from symptomatic LTxRs (n=27), symptomatic (n=6) and asymptomatic (n=57) patients waiting for LTx) were characterized for SARS-CoV-2 spike protein S2. Exosomes from n=7 SARS-CoV-2 from symptomatic LTxRs and n=7 asymptomatic patients waiting for LTx were characterized for other proteins *i.e* SARS-CoV-2 Nucleocapsid NFkB, MST1, GRANZYME Band AGTR1.

The membrane was blocked with 5% BSA and was probed with exosome-specific marker CD9 (312102, BioLegend), SARS-CoV-2 spike protein S2 (Thermofisher Scientific), NFkB (Cell Signaling Technologies), and MST1, GRA-B (Cell Signaling Technologies), AGTR1 (Sigma Aldrich), coronavirus 229E (Novus Biologicals), SARS-CoV-2 nucleoprotein Ab (Pro Science), Col-V (Abcam), Kα1T (Santa Cruz) were used as primary Abs; secondary Abs conjugated with horseradish peroxidase specific to primary Abs were employed. The blots were washed with PBS Tween (Thermo Fisher Scientific), developed using chemiluminescent HRP substrate (WBKLS0500, Millipore Sigma), and exposed using Odyssey CLx Imaging System (LICOR Biosciences). The band intensity of the target protein was quantified using ImageJ software and normalized with CD9.

**1.4 Statistical analysis**

Data analysis was performed using Prism 8.0 software (GraphPad, Inc.). Optical density of exosomes containing lung SAgs was measured using unpaired student’s t-test. P values <0.05 were considered statistically significant in each comparative analysis. The mean optical density of exosomes containing lung SAgs and viral antigens were calculated after normalization with exosome-specific marker CD9 and comparative analysis was performed using non-parametric, Mann-Whitney Test. Statistical significance of the change in exosome protein abundance between each pairwise analysis (control *vs* SARS-CoV-2 either symptomatic or asymptomatic groups for 6 exosome proteins, i.e (AGTR1, MST1, GRA-B, NFkB, Kα1T and Col-V) was determined using Student’s t-test. A Benjamini-Hochberg testing at FDR 5% for multiple corrections was also calculated. The adjusted p values after multiple testing is given in **Tables S2A, B.**

**1.5 Mass spectroscopy of SARS-CoV-2 band from exosomes**

One sample from symptomatic and asymptomatic patients waiting for LTx were taken for mass spectroscopy analysis of exosome protein. Exosomal protein on SDS PAGE stained with coomassie dye and destained. Desired size gel pieces were sliced from gel; protein was extracted using the protocol by Shevchenko A. et al.^1^

**1.6 RT-PCR**

RNA was isolated from exosomes isolated from LTxRs-COVID-19 patients (n=7), Asx, (n=7) and controls (n=5) using QIAamp Viral RNA kit (Qiagen). RT-PCR was performed using the CDC-recommended cDNA synthesis kit (Promega GoTaq Probe 1 step RT-qPCR system) with the CDC-approved primers for qPCR Probe Assay/Primer mix.^2^ RT-PCR was done at settings per instructions provided in the CDC 2019-Novel Coronavirus (2019-nCoV) RT-PCR Diagnostic Panel. Data analysis was done using StepOne software (Applied Biosystems). Data analysis was performed using Prism 8.0 software (GraphPad, Inc.). Comparative analysis was performed using non-parametric, Mann-Whitney Test.

**1.7 Electron microscopy**

Exosomes from control (n=1), symptomatic patients (n=1) and asymptomatic patients waiting for LTx (n=1) were taken for TEM. Samples were immunolabeled with 1:250 dilutions of anti-spike protein S2 and anti-nucleocapsid followed by negative staining for analysis by electron microscopy. Suspensions containing exosomes were allowed to absorb to freshly glow-discharged nickel grids for 10-minutes and then immunolabeled with the primary Abs (anti-spike protein and anti-nucleocapsid) at 1:250 dilution and secondary Ab to goat anti-mouse (IgG and IgM [H & L]) conjugated to 12nm colloidal gold at 1:30 dilution. Grids containing samples were fixed with 1% glutaraldehyde, washed with dH2O, and negative stained with 1% aqueous uranyl acetate.^3^

**1.8 Measurement of circulating cytokines**

Plasma samples were used to measure cytokines by a 25-plex Human Magnetic Luminex assay kit (Invitrogen) according to the manufacturer’s protocol. Briefly, 50µl of plasma (1:50 dilution) was added to the multiplex beads coated in the wells and incubated for 2-hours at room temperature. Cytokines with known concentrations were used as standards. The cytokines bound in the beads were detected using biotinylated anti-human multicytokine reporter and streptavidin-PE detection. The plate was washed and read using BioPlex Luminex (BioRad) and cytokine concentrations were measured using a standard curve (mean fluorescence intensity).^4^ Data analysis was performed using Prism 8.0 software (GraphPad, Inc). Comparative analysis was performed using non-parametric, Mann-Whitney Test.

**1.9 Immunization of mice with exosomes with SARS-CoV-2 antigen**

C57BL/6 mice were used for immunization with exosomes isolated from LTxRs-COVID-19 patients. Animals were immunized without adjuvants: (1) Control group of animals (n=3) with exosome isolated from control without SARS-CoV-2 infection. (2) Animals were immunized (n=6) with exosomes carrying SARS-CoV-2 spike protein isolated from LTxRs with diagnosed SARS-CoV-2 infection. (2) Animals were immunized (n=6) with exosomes carrying SARS-CoV-2 spike protein isolated from Asx patients waiting for transplant who were PCR negative for SARS-CoV-2. Immunizations were carried out subcutaneously with exosomes (100µg per injection on days 1, 7 and 21) without any adjuvant. Blood samples were collected at day 28 following immunization to determine the development of Abs specific to SARS-CoV-2 spike protein by ELISA.

**1.10 Detection of Abs to** SARS-CoV-2 **spike protein**

Development of Abs to SARS-CoV-2 spike antigen was determined using ELISA**.** In brief, 1μg/mL of SARS-CoV-2 spike proteins (Sino Biologicals) suspended in PBS were coated onto an ELISA plate and incubated overnight at 4°C. Mouse serum was added to these plates in serial dilutions pre- and post-immunization (day 28). Detection was performed using secondary anti-mouse immunoglobulin G-horseradish peroxidase (1:10,000) and developed using tetramethylbenzidine substrate and read at 450nm. Antibody concentration was calculated using a standard curve from known concentrations of respective Abs (Santa Cruz Biotechnology). Data analysis was performed using Prism 8.0 software (GraphPad, Inc.). Comparative analysis was performed using non-parametric, Mann-Whitney Test.

**References:**

1. Shevchenko A, Tomas H, Havlis J, Olsen JV, Mann M. In-gel digestion for mass spectrometric characterization of proteins and proteomes. *Nature protocols*. 2006;1(6):2856-60. doi:10.1038/nprot.2006.468
2. Vogels CBF, Brito AF, Wyllie AL, et al. Analytical sensitivity and efficiency comparisons of SARS-CoV-2 RT-qPCR primer-probe sets. *Nat Microbiol*. Oct 2020;5(10):1299-1305. doi:10.1038/s41564-020-0761-6
3. Gunasekaran M, Xu Z, Nayak DK, et al. Donor-Derived Exosomes With Lung Self Antigens in Human Lung Allograft Rejection. *Am J Transplant*. Feb 2017;17(2):474-484. doi:10.1111/ajt.13915
4. Mehta P, McAuley DF, Brown M, et al. COVID-19: consider cytokine storm syndromes and immunosuppression. *Lancet*. Mar 28 2020;395(10229):1033-1034. doi:10.1016/S0140-6736(20)30628-0

**Figure S1:** Nanosight images of representative exosomes from LTxRs-COVID-19 and Asx patients.

| **Table S1: Demographic of 27 LTxRs^†^-COVID-19, 6 symptomatic patients waiting for LTx^‡^ and 16 Asx patients waiting for LTx** | | |
| --- | --- | --- |
| **Variable** | **n** | **Time of Sample Collection** |
| **Control RT PCR negative** | 5 | No infection |
| **Post LTx SARS-CoV-2 Positive LTxRs (n=27) RT PCR positive** | | |
| Mean age, years ± SD | 54.9±16.87 | — |
| Male sex | 17/27 | — |
| Female sex | 10/27 | — |
| Caucasian | 25/27 | — |
| Hispanic | 2/27 |  |
| Bilateral LTx | 27/27 | — |
| **Clinical Condition** |  |  |
| Idiopathic Pulmonary Fibrosis | 6/27 | At the time of SARS-CoV-2 infection |
| Chronic Obstructive Pulmonary Disease | 12/27 | At the time of SARS-CoV-2 infection |
| Interstitial Lung Disease | 3/27 | At the time of SARS-CoV-2 infection |
| Idiopathic Pulmonary Haemosiderosis | 4/27 | At the time of SARS-CoV-2 infection |
| **Patients Waiting for Lung Transplant and Symptomatic (n=6) RT PCR positive** | | |
| Mean age, years ± SD | 42.4±15.81 |  |
| Male sex | 5/6 |  |
| Female sex | 1/6 |  |
| Caucasian | 4/6 |  |
| African American | 1/6 |  |
| Asian | 1/6 |  |
| Bilateral LTx |  | All Waiting at the time of infection |
| ARDS | 1/6 | At the time of SARS-CoV-2 infection |
| Pneumonia | 4/6 | At the time of SARS-CoV-2 infection |
| IPF | 1/6 | At the time of SARS-CoV-2 infection |
| **Patients Waiting for Lung Transplant and Asymptomatic (n=16) RT PCR negative** | | |
| Mean age, years ± SD | 63.0±12.83 |  |
| Male sex | 9/16 |  |
| Female sex | 7/16 |  |
| Caucasian | 14/16 |  |
| Hispanic | 2/16 |  |
| Bilateral LTx |  | Waiting for Lung transplant |
| **SARS-CoV-2 Positive** | | |
| Idiopathic Pulmonary Fibrosis | 5/16 | Not Diagnosed SARS-CoV-2+ by PCR |
| Cystic Fibrosis | 1/16 | Not Diagnosed SARS-CoV-2+ by PCR |
| Pulmonary Fibrosis | 1/16 | Not Diagnosed SARS-CoV-2+ by PCR |
| Chronic Obstructive Pulmonary Disease | 5/16 | Not Diagnosed SARS-CoV-2+ by PCR |
| Interstitial Lung Disease | 1/16 | Not Diagnosed SARS-CoV-2+ by PCR |
| Others | 3/16 | Not Diagnosed SARS-CoV-2+ by PCR |
| ^†^LTxRs – lung transplant recipients; ^‡^LTx – lung transplant | | |

| **Table S2A: Fold change with p values of exosome proteins from SARS-CoV-2 LTxRs^†^-COVID-19, SARS-CoV-2 waiting for LTx^‡^ and Asx plasma as compared to controls.** | | | | | | | | |
| --- | --- | --- | --- | --- | --- | --- | --- | --- |
| **Exosomal Proteins** | | | **Symptomatic Fold Change** | | **p values**  **Compared to Control** | | **P Adj** | |
| SARS-CoV-2 spike protein (n=27 Post LTxRs, Symptomatic) | | | 23.77 | | 0.0007 | | 0.0021 | |
| SARS-CoV-2 spike protein (n=6 Pre LTxRs, Symptomatic) | | | 27.25 | | 0.0238 | | 0.0238 | |
| SARS-CoV-2 spike protein (n=16 Pre LTxRs, Asymptomatic) | | | 25.87 | | 0.0021 | | 0.0238 | |
| **Table S2B: Fold change with p values of exosomal proteins from SARS-CoV-2 LTxRs-COVID-19 (n=7) and Asx plasma as compared to controls (n=7).** | | | | | | | | |
| **Exosomal Proteins** | **Symptomatic Fold Change** | **p values**  **Compared to Control** | **P Adj** | **Asymptomatic Fold Change** | | **p values**  **Compared to Control** | | **P Adj** |
| AGTR1 | 19.14 | 0.0238 | 0.0238 | 18.44 | | 0.0167 | | 0.0221 |
| MST1 | 15.35 | 0.0167 | 0.0238 | 6.79 | | 0.0189 | | 0.0221 |
| Granzyme B | 11.92 | 0.0211 | 0.0238 | 5.56 | | 0.0171 | | 0.0221 |
| Collagen V | 1.05 | 0.0167 | 0.0238 | 17.20 | | 0.0210 | | 0.0221 |
| Kα1 Tubulin | 19.75 | 0.0191 | 0.0238 | 3.47 | | 0.0221 | | 0.0221 |
| NFkB | 7.80 | 0.0171 | 0.0238 | 5.85 | | 0.0166 | | 0.0221 |
| ^†^LTxRs – lung transplant recipients; ^‡^LTx – lung transplant | | | | | | | | |
