# Supplementary figures and images for: SARS‐CoV‐2 infection in lung transplant recipients induces circulating exosomes with SARS‐CoV‐2 spike protein S2
Source: Clin Transl Med. 2021 Nov 4;11(11):e576. doi: 10.1002/ctm2.576 (PMC8567032; doi:10.1002/ctm2.576)

**Symptomatic SARSCoV2 positive sample**

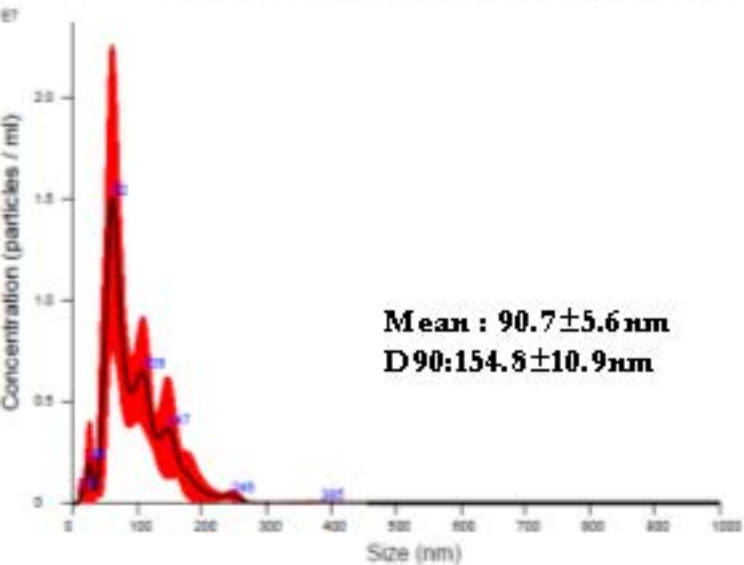

**Asymptomatic SARSCoV2 positive sample**

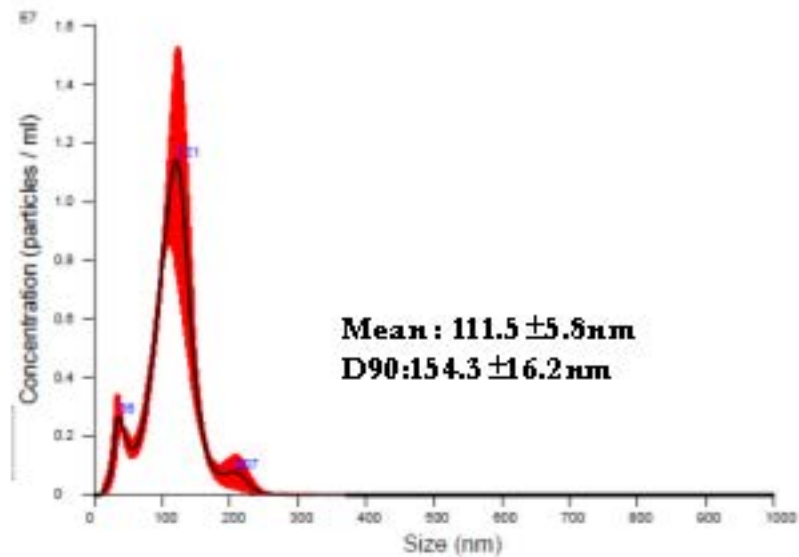

Supplement: Supplementary file 2 — Supporting Information [file CTM2-11-e576-s001.pdf]
